# Comparing Peristaltic and Direct-Drive Contrast Injection Systems for Thoracic Computed Tomography (CT): Effects on Dose, Image Quality, and Pathology

**DOI:** 10.7759/cureus.76814

**Published:** 2025-01-02

**Authors:** Charbel Saade, Suzanne Saab, Jana El-Sakakini, Lina Karout, Carlos Casillas, Edward Chan, Maria El Homsi, Rida Salman, Carlos Nicolou, Lena Naffaa

**Affiliations:** 1 Medical Imaging Sciences, American University of Beirut Medical Center, Beirut, LBN; 2 Radiology, Hospital Vithas Nisa Rey Don Jaime, Castellon, ESP; 3 Radiology, Liverpool Hospital, New South Wales, AUS; 4 Diagnostic Radiology, American University of Beirut Medical Center, Beirut, LBN; 5 Radiology, Hospital Clinic de Barcelona, Barcelona, ESP; 6 Radiology, Nemours Children's Hospital, Orlando, Florida, USA

**Keywords:** adult, chest, computed tomography, contrast media, x-ray

## Abstract

Introduction

To compare thoracic vasculature opacification between the direct-drive and the peristaltic drive contrast injection systems and the effect on quantitative and qualitative image quality during computed tomography.

Method

A retrospective chart review of 88 patients who underwent chest computed tomography (CT) following a direct-drive injector before 2016 (Group A) or a peristaltic injector after 2016 (Group B). Both protocols employed 80mL of iodinated contrast media volume. Quantitative measurements of the thoracic vascular opacification, contrast-to-noise ratio (CNR), effective radiation dose, and arterial venous contrast ratio (AVCR) for each vessel were computed and compared. The measured values of vascular opacification, CNR, and dose length product (DLP) were assessed utilizing paired t-test and Pearson’s correlation. Receiver operating characteristic (ROC), visual grading characteristics (VGC), and Cohen’s kappa methodology were measured.

Results

There was no significant difference in the mean opacification of the chest vasculature (p > 0.05). The CNR in Group B was significantly lower than in Group A (p < 0.05) except for the brachiocephalic artery, proximal descending aorta, and the right pulmonary artery. Arterial venous contrast ratio at all anatomic levels demonstrated no statistical significance between groups. There were no differences in ROC, VGC, or kappa values between groups.

Conclusion

When comparing two contrast media injection systems in chest CT, there was no qualitative difference in image quality or pathology detection. However, quantitatively, direct-drive injectors provided higher CNR. Direct-drive contrast injectors also provided a superior quantitative image quality of chest vasculature compared to peristaltic injectors.

## Introduction

Optimal thoracic vascular and parenchymal opacification during computed tomography (CT) is influenced by different contrast media administration techniques such as a single bolus in contrast with split bolus injection techniques with or without a saline chaser [1]. There have been several attempts to improve the quantitative and qualitative image quality of chest CT examinations when employing various radiation dose reduction and contrast media administration strategies [1-8]. More elaborate work has been conducted on the radiation dose among different types of CT studies due to the increased risk of developing cancer. The estimated number of CT scans that will lead to such a side effect depends on the type of CT examination and the patient's age and sex, being higher in the younger age group and more complicated studies such as multiphase CT scans [9]. Furthermore, more specific research work studied the effect of iodinated contrast agents during imaging procedures, such as chest CT, demonstrated a significant increase in the level of radiation-induced DNA damage by increasing the average number of phosphorylated histone (γH2AX) foci per peripheral blood lymphocyte [10]. The establishment of clinically acceptable image quality is a complex task engaging objective quantitative measures such as contrast-to-noise ratio (CNR) and until recently, arteriovenous contrast ratio (AVCR) [1]. Qualitative image analysis was performed by employing visual grading characteristics (VGC) and Cohen’s kappa values [11]. As a distinguishing factor in CT imaging, contrast media administration plays a paramount role in both qualitative and quantitative assessment of image quality and pathology detection [12]. Finally, to our knowledge, there is no existing literature focusing on image quality measured quantitatively and the viable clinical indicators between two main types of contrast media injections (direct-drive and peristaltic).

To date, there have been no known studies employing peristaltic pump injection systems on humans. As such, there are no studies comparing peristaltic to direct-drive injectors during chest CT. These two types of contrast media delivery systems differ significantly in their mechanical properties [1-3]. The first system consists of a direct-drive contrast injector-which is made of a piston plunger with a ram moved by a drive motor, an elastic plunger, and a stretcher that fills a syringe as a reservoir to inject contrast media into the patient [2, 3]. The second system consists of a peristaltic drive contrast injector, which employs compression and relaxation of the tube to draw the contents (contrast media and saline) into the delivery tube; this creates a seal between the suction and discharge side of the pump, eliminating product slip and reducing delivery pressure of the contrast media into the injected vein [1-3]. Technically, there are differences between these two injection systems with respect to achieving higher flow rates and varying saline or contrast media concentrations upon delivery [6]. In this study, we discuss and compare the two main injection techniques considering vascular opacifications in the thorax and the outcomes of qualitative and quantitative image quality while highlighting the total radiation dose to the patient.

## Materials and methods

This retrospective study was approved by the Institutional Review Board of the American University of Beirut. As part of the approval, patient consent was waived. Before 2016, a direct-drive pump contrast media injector was employed at our university hospital. After 2016, the injector was substituted with a peristaltic contrast media with iodinated contrast media.

Study population 

A total of 88 patients who underwent CT scans of the chest with iodinated contrast media utilizing a direct drive pump injector (before 2016) and later on utilizing the peristaltic pump injector (after 2016) were included in the study. This same group of patients was named Group A or Group B depending upon which injection technique they received. Group A (88 patients) received the direct-drive injection method (Optivantage, Guerbet, France), and Group B (88 patients) received the peristaltic injection method (CT Motion, Ullrich, Germany).

CT scanning protocol

Computed tomography was performed using a 256-channel scanner (Philips Brilliance iCT, Philips Healthcare, Netherlands). Both contrast media groups employed a tube voltage of 100 kVp and 180 mAs with x, y, and z-axis modulation (DoseRight, Philips, Netherlands). The temporal resolution was 0.3 sec/rot with a pitch value of 1.375 mm/rot. The detector width was 256 x 0.625 mm.

Contrast media administration

The contrast media volume for Group A (direct-drive) was 80 mL of Ioversol (Optiray 350 mgI/mL; Guerbet, France). For Group B (peristaltic drive), the contrast media volume was 80 mL of Iohexol (Omnipaque 350 mgI/mL; GE Healthcare, USA). Both groups received the injection intravenously at a flow rate of 3 mL/s with a 100 mL saline flush. Each acquisition had a 60-second post-injection delay (venous phase) for all routine imaging.

Quantitative image assessment

Chest images were assessed in the venous phase of the chest CT for the mean arterial, venous lymph node and soft tissue structures. A slice thickness of 3 mm was used for all chest exams to measure the opacification profile of the thoracic vasculature and the CNR for each arterial (n = 18) and venous (n = 9) segments.

Image reconstruction

Trans-axial images were reconstructed with a model-based iterative reconstruction algorithm (iMR, Philips Healthcare, Netherlands). All images employed iMR, Level 2. Images were reconstructed using 3 x 3 mm slice thickness using a smoothing convolution kernel (field of view 380 x 380 mm; image matrix 512 x 512).

CNR measurement

The CNR was calculated by measuring the mean thoracic vasculature Hounsfield Unit (HU) and standard deviation. The CNR = μA - μB / σA, where μA is the mean region of interest (ROI) value for each of the vessels, μB is the mean ROI value of the HU of air, and σA is the standard deviation of the ROI values for the total vasculature.

Radiation dose measurement

For each of the CT scans, individual effective dose [Eff (mSv)] was calculated from the dose-length products [DLP (mGy×cm)], which were recorded from the patient groups. A normalized conversion factor [k (mSv/mGy×cm)} for the chest (mSv/mGy×cm) was used to calculate the Eff (1): Eff = DLP x k.

Qualitative image assessment

The image observer bank consisted of 88 patients who had a chest CT with a direct-drive contract injector before 2016 (Group A) and their corresponding chest CT with peristaltic contrast injector type after 2016 (Group B), totaling 176 images. The images were randomly arranged and blinded, with the contrast injection group not revealed to the observers. All images were assessed in a single sitting by nine radiologists who are experts in chest CT with more than 10 years of reading experience in various institutions around the world (multicenter reader observer).

Receiver operating characteristic analysis 

Receiver operating characteristic (ROC) methodology was employed to illustrate radiologist confidence intervals to detect pathology. A score of 1 or 2 was assigned to each image of chest lesions, where 1 indicated positive for pathology detection, and 2 indicated negative for pathology detection.

VGC analysis

The VGC method was employed to illustrate radiologist preference for one injection group over another based on qualitatively assessing image quality. A score between 1 and 5 was assigned- where 1 indicated poor image quality, and 5 indicated optimal image quality for each anatomical structure including arterial, venous, lymph node, and soft-tissue structures, as well as overall image quality for diagnosis.

Inter- and intra-reader variability

In each group, the inter- and intra-observer agreements were calculated using Cohen kappa analysis. A kappa value of 0.60 to 1, 0.41 to 0.60, 0.21 to 0.40, or less than 0.20 were considered excellent, moderate, fair, or poor agreement, respectively.

Statistical analysis

Vascular opacification and radiation dose measurements were compared employing paired t-test and Pearson's correlation. Results were considered statistically significant if p ≤ 0.05. Categorical variables were presented as frequencies with percentages, and continuous variables were presented as means ± standard deviations. ROC and VGC were employed to measure the confidence intervals in pathology detection and image quality, respectively. Inter- and intra-observer variations were investigated employing Cohen’s kappa methodology.

## Results

Quantitative analysis

Patient Demographics

All patients were White, with a mean age of 48.6 ± 20.89 for Group A and 49.02 ±20.87 for Group B. A significant age difference was found between the two groups (p < 0.0001) due to the time between the first scan and the second scan of the same patient averaging 0.4 year. No significant difference was found among sexes between the two groups.

Opacification of Thoracic Vasculature

There was no significant difference in the mean opacification of the arterial vasculature of the chest (p > 0.05). Additionally, no significant difference was found in the mean opacification of the venous vasculature (p > 0.05) except for the right internal jugular vein, which had a more statistically significant opacification in Group B (p = 0.040; Table 1).

**Table 1:** Mean vascular opacification of the chest and its vasculature following either direct-drive (Group A) or peristaltic drive (Group B) injector protocol. Data are displayed as mean ± standard deviation in Hounsfield units. Statistical significance is defined when the p value is less than 0.05.

	Group A Direct-Drive Injector	Group B Peristaltic Drive Injector	p value
Arterial			
Right Carotid Artery	230.55 ± 73.89	245.06 ± 86.89	0.243
Left Carotid Artery	230.55 ± 73.89	242.63 ± 79.39	0.250
Right Subclavian Artery	206.09 ± 79.15	220.47 ± 95.07	0.270
Left Subclavian Artery	198.85 ± 88.82	217.62 ± 240.89	0.500
Brachiocephalic Artery	235.81 ± 75.19	295.19 ± 453.59	0.230
Ascending Aorta 1	234.95 ± 73.85	250.76 ± 103.97	0.242
Ascending Aorta 2	237.87 ± 76.02	243.39 ± 83.38	0.627
Transverse Aorta 1	238 ± 77.24	251.28 ± 87.78	0.250
Transverse Aorta 2	235.69 ± 71.78	246.53 ±85.98	0.328
Transverse Aorta 3	258.21 ± 224.52	247.93 ± 79.97	0.691
Descending Aorta 1	234.55 ± 75.17	298.17 ± 515.53	0.246
Descending Aorta 2	226.97 ± 75.16	237.26 ± 86.11	0.352
Descending Aorta 3	231.05 ± 76.97	241.83 ± 86.5	0.334
Pulmonary Trunk	220.55 ± 68.56	228.91 ± 63.26	0.315
Right Pulmonary Artery	213.87 ± 65.05	219.81 ± 60.23	0.463
Left Pulmonary Artery	213.41 ± 68.56	223.95 ± 61.07	0.199
Distal Right Pulmonary Artery	218.55 ± 78.01	218.52 ± 72.87	0.997
Distal Left Pulmonary Artery	215.69 ± 77.89	225.79 ± 80.14	0.346
Venous			
Right Subclavian Vein	360.72±332.42	345.48±340.75	0.740
Left Subclavian Vein	285.89±390.02	273.83±310.72	0.790
Right Brachiocephalic Vein	516.09±424.07	475.69±365.89	0.465
Left Brachiocephalic Vein	320.28±362.60	337.64±370.48	0.750
Superior Vena Cava	405.65±237.43	388.91±180.39	0.593
Azygous Vein	103.26±39.95	112.64±59.10	0.179
Right Internal Jugular Vein	201.99±73.97	232.44±123.13	0.040
Left Internal Jugular Vein	203.85±71.34	215.61±92.78	0.274
Inferior Vena Cava	132.87±47.14	131.51±40.39	0.790

CNR

When comparing the opacification of the chest vasculature (arteries and veins), the CNR in Group B was significantly lower than Group A (p < 0.05) except for the brachiocephalic artery, proximal descending aorta, and the right pulmonary (Table 2).

**Table 2:** Contrast-to-noise ratio for each vascular structure in the chest following either direct-drive (Group A) or peristaltic drive (Group B) injector protocol. Data are displayed as mean ± standard deviation in Hounsfield units. Statistical significance is defined as when p value is less than 0.05.

	Group A Direct-Drive Injector	Group B Peristaltic Drive Injector	p value
Arterial			
Right Carotid Artery	178.48 ± 107.58	139.90 ± 111.34	0.025
Left Carotid Artery	178.34 ± 107.51	138.95 ± 109.48	0.021
Right Subclavian Artery	174.24 ± 104.83	136.20 ± 106.93	0.022
Left Subclavian Artery	173.29 ± 104.8	135.71 ± 107.66	0.021
Brachiocephalic Artery	178.74 ± 107.58	149.78 ± 154.33	0.177
Ascending Aorta 1	178.88 ± 107.62	140.12 ± 110.67	0.023
Ascending Aorta 2	179.41 ± 108.01	138.73 ± 109.35	0.017
Transverse Aorta 1	179.38 ± 108.06	140.19 ± 111.37	0.023
Transverse Aorta 2	179.10 ± 107.88	139.66 ± 110.79	0.021
Transverse Aorta 3	182.49 ± 112.54	129.94 ± 111.35	0.016
Descending Aorta 1	178.85 ± 107.61	153.96 ± 195.26	0.292
Descending Aorta 2	177.87 ± 107.26	138.60 ± 110.09	0.021
Descending Aorta 3	178.49 ± 107.60	139.17 ± 110.48	0.022
Pulmonary Trunk	176.51 ± 106.28	137.04 ± 106.76	0.018
Right Pulmonary Artery	213.87 ± 65.05	219.81 ± 60.23	0.463
Left Pulmonary Artery	175.95 ± 106.33	136.17 ± 105.83	0.017
Distal Right Pulmonary Artery	175.01 ± 105.33	135.32 ± 105.71	0.018
Distal Left Pulmonary Artery	175.68 ± 105.63	137.13 ± 107.56	0.021
Venous			
Right Subclavian Vein	193.13 ±122.66	147.83 ±123.41	0.011
Left Subclavian Vein	189.17 ± 131.15	143.08 ±117.61	0.012
Right Brachiocephalic Vein	212.92 ±140.05	162.61 ±139.27	0.016
Left Brachiocephalic Vein	194.42 ± 130.53	148.46 ± 117.33	0.019
Superior Vena Cava	199.93 ± 119.78	154.08 ± 122.09	0.015
Azygous Vein	160.05 ± 97.70	123.91 ± 96.31	0.018
Right Internal Jugular Vein	173.46 ± 104.71	136.38 ± 106.63	0.026
Left Internal Jugular Vein	174.56 ± 105.25	135.87 ± 107.57	0.020
Inferior Vena Cava	164.85 ± 101.20	125.47 ±96.82	0.012

AVCR

The AVCR at the supra-aortic level was 1:13 in Group A and 1:0.99 in Group B. At the cervicothoracic junction, Group A was 1:0.97 and Group B was 1:72. Group A was 1:0.93 at the aortic level, while Group B was 1:0.99. At the infra-annulus, Group A was 1:1.05 and Group B 1:13. The difference failed to be significant at all the levels (Table 3).

**Table 3:** Arteriovenous contrast ratio for each anatomical level in the chest. Data are mean ± standard deviation in Hounsfield units. Statistical significance is defined as when p value is less than 0.05.

	Group A Direct-Drive Injector	Ratio	Group B Peristaltic Drive Injector	Ratio	p value
Supra-Aortic					
Right/left Carotid Arteries	230.55 ± 73.89	1: 1.13	243.85 ± 83.14	1:1.09	>0.05
Right/left Jugular Vein	202.92 ± 72.66		224.03 ± 107.96		
Cervical-Thoracic Junction					
Right/Left Subclavian & brachiocephalic Artery	213.58 ± 81.05	1:0.97	224.43 ± 263.18	1:0.72	>0.05
Right/left Subclavian Vein	323.31 ± 361.22		309.66 ± 325.74		
Supra-Aortic					
1/2 Ascending & Transverse 1 Aorta	236.94 ± 75.70	1:0.93	248.48 ± 91.71	1:0.99	>0.05
Superior Vena Cava/Azygous Vein	254.46 ± 138.69		250.78 ± 119.75		
Infra-Annulus					
Descending 1/Descending 2/Descending 3 Aorta	230.86 ± 75.77	1:1.05	259.09 ± 229.38	1:13	>0.05
Pulmonary Trunk	220.55 ± 68.56		228.91 ± 63.26		

Radiation Dose

The radiation dose in Group B was lower than in Group A; however, we did not observe statistical validity (p = 0.106; Table 4).

**Table 4:** Radiation dose measurements between contrast injector groups. Data are mean ± standard deviation in mGy and mSv. Statistical significance is defined as when p value is less than 0.05.

	Group A Direct-Drive Injector	Group B Peristaltic Drive Injector	p value
Dose length product (mGy)	362.79 ± 146.97	329.29 ±131.52	0.106
Effective dose (mSv)	6.531 ± 2.65	5.9382 ± 2.37	0.106

Qualitative image evaluation

Visual Grading Characteristic

The five-point scores were individually graded by eight radiologists for each protocol employing the Likert scale. There was no statistical significance between arterial opacification, venous opacification, lymph node delineation, soft tissue delineation, and overall image quality to dose between Group A (VGC AUC: 0.35-0.44) when compared with Group B (VGC AUC: 0.36-0.45) (p < 0.465).

ROC

**Figure 1:**
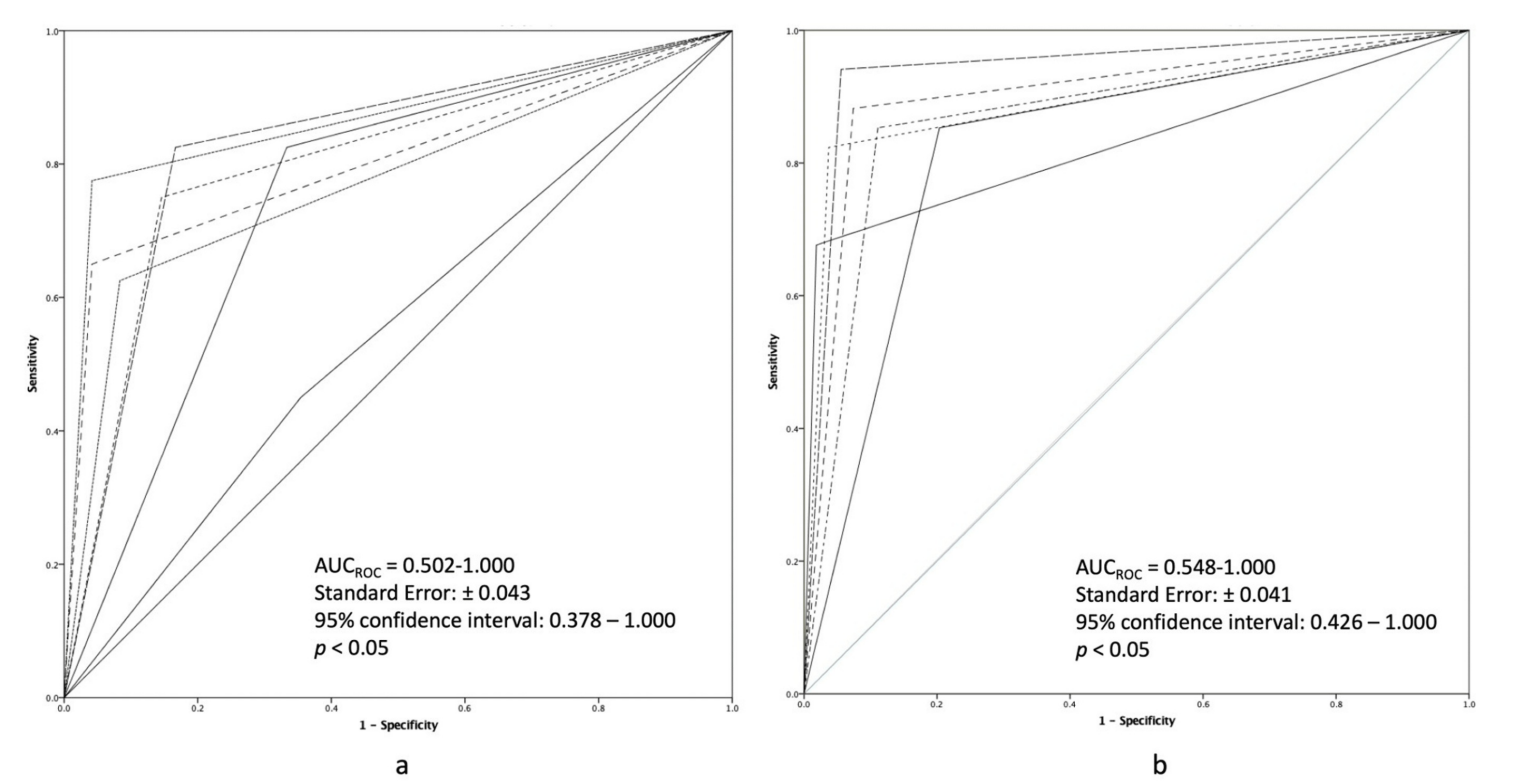
Receiver operating characteristic curve analysis for pathology detection in (a) direct-drive (Group A) and (b) peristaltic drive (Group B) injection protocol.

Detection of thoracic lesions (including lymph nodes) demonstrated a significant difference (p < 0.05) between positive and negative for pathology in both groups. However, when both groups were compared, the confidence interval for radiologists (AUC) demonstrated no statistical significance (Figure 1).

Cohen Kappa Analysis

The inter-and intra-reader agreement was evaluated between the two groups. Statistical analysis showed no significant difference in arterial opacification (p < 0.390) or venous opacification (p < 0.427) between Group A (aortic: 0.02-0.297; venous: -0.065-0.313) and Group B (aortic: -0.022-0.386; venous: -0.037-0.315). No statistically significant difference was seen in lymph node delineation (p < 0.448) or soft-tissue delineation (p < 0.477) between Group A (lymph node: kappa= -0.059-0.308; soft-tissue: kappa = -0.059-0.316) and Group B (lymph node: kappa = -0.075-0.252; soft-tissue: kappa = -0.007-0.294) (P < 0.021). Finally, there was no difference in agreement when evaluating the overall image quality to diagnose between Group A (-0.076- 0.339) and B (-0.034-0.297) (p < 0.5).

## Discussion

In this study, we compared several different quantitative and qualitative factors regarding thoracic vasculature between the peristaltic and direct-drive contrast media injection methods. Different quantitative approaches were utilized in our research. We evaluated the opacification levels, CNR, and AVCR of the thoracic vasculature. Consequently, a qualitative assessment was employed using VGC, ROC, and kappa analysis. The results revealed neither injection system had any difference in terms of mean vascular opacification, except for the right internal jugular vein which was statistically significant (p < 0.05). Further into our quantitative analysis, there was a significant drop in CNR values when employing the peristaltic contrast media injector. The CNR values for group B were significantly lower than those for group A (p < 0.05), which could account for the non-uniform contrast delivery in the peristaltic approach when compared with the direct-drive approach [6]. This could potentially highlight the need for future studies aiming to reduce contrast volume when employing direct-drive injectors. While we measured the levels of the mean vascular opacification between the direct-drive and peristaltic injection systems, CNR and AVCR, we observed a decreased CNR in Group B, indicating the decline of quantitative image quality.

**Figure 2:**
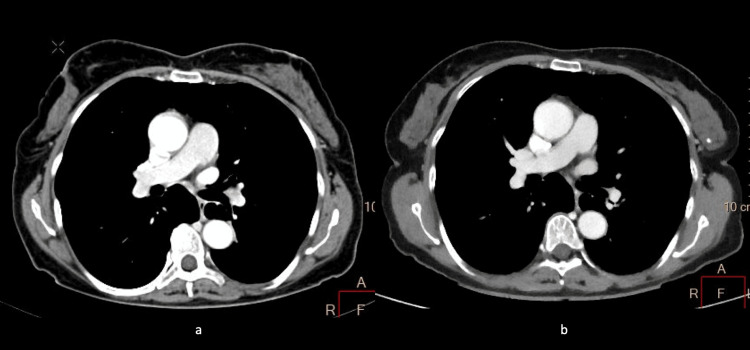
A 52-year-old female presenting with routine imaging for suspected chest malignancy who underwent (a) direct-drive (Group A) and (b) peristaltic drive (Group B) contrast media injection protocol within three months of each other.

Contrary to the results obtained by the quantitative tests, the utilization of qualitative assessments (VGC, ROC, and kappa analysis) allowed us to conclude that neither injection system had any significant difference in the diagnostic value or pathology detection as assessed by international reviewers (multi-center), and thus, no difference was found in the qualitative measurement of image quality. For VGC, no significant difference was reported between the two groups (Figure 2). Although a difference between positive and negative findings regarding pathology in both groups was perceived, with regards to ROC analysis when both groups were compared, the AUC demonstrated no statistical significance.

Group B images, on average, were taken six months after the images were acquired for group A. Since there was no change in pathology detection between the groups, and we have confirmed there was no difference in qualitative image quality, there could or could not have been an increase in the pathology of the patient. Otherwise, the patients who were chosen would not have had any pathology pertaining to oncology where changes can be seen in short periods of time.

Patients who undergo constant CT examinations are subjected to high amounts of radiation [9, 10]. On this account, it is crucial to decrease the radiation dose whenever possible. There also would be reduced accessory costs when employing multi-use system savings as well as the potential reduction of radiation doses compared to direct-drive injection systems [9, 10]. In this way, the utilization of a peristaltic injection system would greatly contribute to the well-being of the patient in multiple injections over a long period of time for pathology evaluation. Even though we did not achieve significant results quantitatively, there was no difference in the qualitative results between the groups, which signifies that the image quality between groups was the same for radiologists. Moreover, although the radiation dose in peristaltic drive was lower than in direct-drive injection, we did not observe a statistical difference (p > 0.05).

Another study in the literature assessed the diagnostic and physical image quality of the chest throughout different CT image reconstructions [13]. The results demonstrated the importance of being meticulous while measuring the efficiency of CT equipment by only considering quantitative measurements such as CNR [13]. This might give an insufficient depiction of the actual image quality achievement. It highlights the importance of qualitative measures in assessing acquired CT images. Finally, while we did employ model-based iterative reconstruction, this could have shown the reduced image noise due to technology advances and not contrast media delivery [4, 5].

Our study has a few limitations. It is a retrospective analysis of a small sample size. Random errors could have occurred due to human inaccuracy in properly measuring the required values with the same precision. In addition, were not able to calculate SNR because we did not have the standard deviation factors for each vessel. Computing the SNR would have added further support to the data and results of the study and better sustained our study’s hypothesis.

## Conclusions

Our study emphasizes the existence of a broad spectrum of systems to be discovered and validated for chest CT scans. When the two contrast media injection systems in chest CT were compared, there was no difference in image quality or pathology detection utilizing qualitative assessments (VGC, ROC, and kappa analysis). However, upon quantitative assessment of each vessel (thoracic vascular opacification, CNR, and AVCR), direct-drive injectors provided higher CNR and superior quantitative image quality of chest vasculature compared to peristaltic injectors. We were able to extract valuable information from the measured values, even when the image CNR was compromised according to quantitative measurements. The peristaltic injection system of contrast media failed to give us a statistically significant reduction in radiation dose. Our study will shed light upon future examinations needed in contrast-enhanced chest CT scans with a larger sample size for better comparison between the two injection techniques.
